# Bone marrow stem cell dysfunction in radiation-induced abscopal bone loss

**DOI:** 10.1186/s13018-015-0339-9

**Published:** 2016-01-07

**Authors:** Qiong Zou, Wei Hong, Yi Zhou, Qiaoling Ding, Jinfeng Wang, Weifang Jin, Jianjun Gao, Guoqiang Hua, Xiaoya Xu

**Affiliations:** Department of Radiation Biology, Institute of Radiation Medicine, Fudan University, No. 2094 Xie-Tu Rd. Building 1, Room 407, Shanghai, 200032 China; Department of Osteoporosis, Hua Dong Hospital Affiliated to Fudan University, Shanghai, China

**Keywords:** Irradiation, Abscopal effect, Marrow adiposity, BMSCs, Runx2, PPAR γ

## Abstract

**Background:**

Bone-related complications are commonly reported in cancer patients receiving radiotherapy and are collectively referred to as the abscopal effect of irradiation, the mechanism of which remains poorly understood. When patients receive targeted radiotherapy to a tumor, the local skeleton is exposed to radiation, particularly within the bone marrow. We therefore investigated the hypothesis that single bone irradiation can induce deterioration of the skeleton outside the radiation field and is mediated by the bone marrow.

**Methods:**

Using 4-month-old male Sprague-Dawley rats, the effects of irradiation (20 Gy, right distal femur and proximal tibia) on bone quality, microarchitecture and bone marrow, were evaluated prospectively by microcomputed tomography, histomorphometry, real-time polymerase chain reaction, and Western blot analysis.

**Results:**

At 12 weeks post-irradiation, bone loss of the non-irradiated bone was induced and marrow adiposity was increased. Expression of runt-related transcription factor-2 by bone mesenchymal stem cells (BMSCs) decreased after irradiation by 88.0 % (*P* < 0.01) at the contralateral and 82.3 % (*P* < 0.01) at the irradiation site 2 weeks post-irradiation and decreased by 94.5 % (*P* < 0.001) at the contralateral and 44.1 % (*P* < 0.05) at the irradiation site 12 weeks post-irradiation. Interestingly, peroxisome proliferator-activated receptor gamma expression decreased by 61.8 % (*P* < 0.05) at the contralateral and by 48.3 % (*P* < 0.05) at the irradiation site 2 weeks post-irradiation but increased by 9-fold at the contralateral (*P* < 0.001) and by 13-fold (*P* < 0.001) at the irradiation site 12 weeks post-irradiation.

**Conclusions:**

These data highlight that radiation-induced bone complications are partly BMSC-mediated, with important implications for bone health maintenance in patients receiving radiotherapy.

## Background

Following the discovery of X-rays by the German physicist Wilhelm Conrad Roentgen, radiation was used to treat cancer patients and has since been proven as an effective treatment [1, 2]. However, the side effects of irradiation come at the expense of normal tissue injury, especially with the widespread use of radiotherapy in cancer patients [3, 4]. Local irradiation is known to be a common treatment for malignancies such as prostate, pancreatic, cervical, rectal, and endometrial cancers, and with direct irradiation exposure to the bone being inevitable, adverse effects of irradiation on the skeleton are common [5–8]. The frequently reported skeletal complications after local irradiation include regional and systemic osteopenia, osteoporosis, osteonecrosis, and non-malignancy fracture, all of which can seriously reduce the life quality of cancer survivors [9, 10]. Diagnosis of these skeletal complications is often prompted by patient pain at the affected bones. The median time for a fracture diagnosis is between 6 and 16.9 months for female patients treated with radiotherapy for pelvic malignancies when acute syndrome of irradiation is not observed [9, 11, 12]. During this prolonged time after irradiation, the mechanisms by which accumulated changes in the bone occur without irradiation exposure are not clear and appear to be multifactorial. A typical treatment regimen of ionizing radiation for gynecological cancers consists of administration of up to 60 Gy fractionated over a 6-week span [13, 14]. Healthy by-standing tissue, including bone, is estimated to absorb up to half of this dose (~30 Gy) [14, 15]. The effects of radiation on the bone have been typically characterized using total-body irradiation models where widespread systemic inflammation and radiation-induced hypogonadism can complicate data interpretation with regard to the bone. In this present study, we tested the hypothesis that single bone irradiation can induce the deterioration of the skeleton outside the radiation field. Analyses were performed at two time points (2 and 12 weeks) after irradiation.

## Methods

### Ethical approval

The experiment procedure and isolation of primary rat bone mesenchymal stem cells (BMSCs) were carried out in accordance with the Institutional Animal Ethics Committee.

### Animal treatment

Male Sprague-Dawley rats (Shanghai Lab Animal Resource Center, STCSM, Shanghai, China) were used for experiments at 4 months of age. Rats in the irradiated group (*n* = 40) were anesthetized with ketamine and placed into a ^137^Cs γ-ray irradiation chamber and exposed to 20 Gy (0.8 Gy/min for 25 min, ^137^Cs γ-ray irradiation machine) of the right limb of a 2 cm by 2 cm area covering the proximal tibia and distal femur. Non-irradiation body parts including the skeleton were shielded with a custom-made plumbum block and contralateral sides of the femur and tibia (bone which did not receive radiation but was removed from irradiated rats) served as areas distant from irradiation. Control rats (*n* = 40) were similarly manipulated, anesthetized, and underwent sham irradiation (0 Gy). All experiments involving animals were performed according to institutionally approved and current animal care guidelines.

### Micro-CT analysis

Micro-CT analysis of the femur was studied by SkyScan-1176 microcomputed tomography (μCT, Bruker micro CT, Belgium) system. Scans were performed using PANalytical’s Microfocus Tube, 17.93 μm voxel size, 65 KV, 385 μA, and 0.5° rotation step (180° angular range). Micro-CT evaluation of the trabecular bone was performed on a 2-mm region of metaphyseal spongiosa in the distal femur. The regions were located 0.5 mm above the growth plate. Measurements of the cortical bone were performed on a 1-mm region of the middiaphysis of the femur. The 1.6 version of NR econ software was used for 3D reconstruction and viewing of images. After 3D reconstruction, the 1.13 version of CT software was used for bone analysis.

### Bone mineral density analysis

Densitometry was performed by a dual-energy X-ray absorptiometery (DXA) using high-resolution scans on the femur and tibia. In brief, the isolated femurs and tibias were placed on the same location on the platform of a dual-energy X-ray absorptiometer (Discovery A, Hologic Inc., Bedford, MA, USA) and scanned using high-resolution imaging adapted for bone mineral density (BMD) measurement of small animal skeletal subregions according to the manufacturer’s instruction.

### Bone biomechanical quality evaluation

The biomechanical quality was evaluated by the three-point bending test (femur). The tests were performed on an electronic universal material testing machine (INSTRON-5543, USA) using Merlin software according to the manufacturer’s instruction. The maximum load of the femur was obtained automatically from the load-strain curve in the three-point bending test with a span of 18 mm and a loading speed of 10.0 mm/min.

### Histological examination and histomorphometry

The tibia sections were stained with hematoxylin and eosin (H&E), Oil red O, and Mayer’s hematoxylin and histochemically for alkaline phosphatase (ALP) activity using BCIP/NBT kit (Beyotime Biotechnology, China) and tartrate-resistant acid phosphatase (TRAP) activity using TRACP kit (Sigma, USA), respectively. The sections were then counterstained with methyl green and mounted in Kaiser’s glycerol jelly. The following parameters were measured: the ALP-positive osteoblast surface per bone surface (OB.S/BS, %) for bone formation, the TRAP-positive osteoclast surface per bone surface (OC.S/BS, %) for bone resorption, and the adipocyte area per bone marrow area without trabecula (%) for marrow adiposity [16]. Images of micrographs from single sections were digitally recorded using a rectangular template, and recordings were processed and analyzed using Image Pro Plus image analysis software (Image Pro Plus, version 4.112, Media Cybernetics, LP, USA).

### ELISA measurement of bone turnover markers in serum

Blood was collected at 2 and 12 weeks post-irradiation; serum from each rat was analyzed individually in duplicate for the bone formation marker osteocalcin (OCN) using the Rat Osteocalcin EIA Kit (Immunodiagnostic Systems Inc., England); and bone resorption was examined with the marker tartrate-resistant acid phosphatase 5b (TRAP5b) using the Rat TRAP Assay (Immunodiagnostic Systems Inc., England), following the instructions of the manufacturers. The average value of the duplicate measurements was obtained for each rat.

### Cell culture

Bone mesenchymal stem cells (BMSCs) of the tibia and femur 2 and 12 weeks after irradiation were flushed out with a-MEM (Gibco BRL, Carlsbad, CA); cells were seeded on 100 mm culture dishes (Nunc, Rochester, NY) and cultured in l-DMEM supplemented with 100 IU/ml penicillin, 100 mg/ml streptomycin (Gibco BRL), and 10 % fetal bovine serum (FBS, Gibco BRL). Medium was replaced every 3–4 days to remove no-adherent hematopoietic cells. After 2 weeks, the adherent cells were collected and sorted by flow cytometry with CD29, CD90, CD34, and CD45. The sorted BMSCs (CD29+, CD90+, CD34−, CD45−) were cultured in fresh medium and further subcultured. The first passage of sorted BMSCs was termed passage 1. BMSCs between passages 3 and 5 were used for experiments.

### Quantitative real-time PCR

Passage 3 BMSCs were collected, and the total RNA was extracted with Trizol reagent (15596; Invitrogen, Carlsbad, CA) according to the manufacturer’s protocol. Total RNA was reversely transcribed to complementary DNA (cDNA) using the QuantiTect Rev Transcription Kits (205311; Qiagen, Chatsworth, CA). The number of cDNA molecules in the reverse-transcribed samples was determined by real-time PCR analyses using a modified method with QuantiTect SYBR Green PCR Kits (204143, Qiagen) on the Mx3000P Real-Time PCR system (Stratagene, La Jolla, CA). The primers were obtained from SBSgene (www.sbsgene.com) with the following sequences: Runx2, 5′-AGCCTCTTCAGCGCAGTGAC-3′ and 5′-CTGGTGCTCGGATCCCAA-3′ (132 bp, AF187319); PPAR γ, 5′-TCAGGTTTGGGCGAATGC-3′ and 5′-TTTGGTCAGCGGGAAGGA3′ (152 bp, Nm013124.3); and GAPDH,5′-AAACCCATCACCATCTTCCA-3′ and 5′-GTGGTTCACACCCATCACAA-3′ (198 bp, DQ403053), using the protocol as previously described [17]. The conditions were 12.5 μL of master SYBR green I, 0.25 μM of each 5′ and 3′ primer, and 2 μL of samples and/or H_2_O to a final volume of 25 μL. A melting curve was obtained at the end of each run to discriminate specific from nonspecific cDNA products. Content of cDNA was normalized by subtracting the cycle numbers of glyceraldehyde-3-phosphate dehydrogenase (GAPDH) from the target gene (∆Ct = Ct of target gene − Ct of GAPDH), and gene expression level was calculated using 2^−(∆Ct)^.

### Western blotting analysis

Passage 5 BMSCs were collected and lysed with RIPA buffer (P0013B; Beyotime, Jiangsu, China). Cells were extracted for 20 min on ice. Insoluble materials were removed by centrifuging at 12,000rpm for 30 min, the supernatants were collected, and protein amount was then quantified with the BCA protein assay (P0012, Beyotime) using BSA as a standard. The sample protein was denatured in boiling water for 5 min in SDS-PAGE sample loading buffer (P0015, Beyotime). Aliquots of samples (40 μg) were then subjected to SDS-PAGE in 12 % gels under reducing conditions and electroblotted onto PVDF membranes (Ipvh00010; Millipore, Bedford, MA). The membranes were blocked with 5 % fat-free dry milk in tris-buffered saline and tween (TBST) (0.1 % Tween-20 and 0.1 M NaCl in 0.1 M Tris-HCl, pH 7.5) for 2 h at room temperature and with 1:500 goat anti-Runx2 antibody (ab56326; Abcam, Cambridge, MA) or 1:1500 rabbit anti-PPAR γ antibody (AT819, Beyotime) or 1:5000 mouse anti-GAPDH antibody (Kangcheng, Shanghai, China) at 4 °C overnight. Membranes were then incubated with horseradish peroxidase-conjugated secondary antibody (1:5000; Santa Cruz Biotechnology, Santa Cruz, CA) at room temperature for 1 h, followed by chemiluminescence detection (P0018, Beyotime). Each incubation step was followed by three washes (10 min each) with TBST. The protein bands were quantitatively analyzed by using an image analysis system (QuantityOne software; BioRad, Hercules, USA).

### Statistical analysis

Differences were determined by one-way ANOVA, with Bonferroni post hoc testing, or by the paired or unpaired Student *t* test, as appropriate (GraphPad, Prism 6, version 6.0c). Results are expressed as means ± standard deviations, and *P* < 0.05 was considered significant.

## Results

### Bone microarchitecture changed after single radiation

**Fig. 1:**
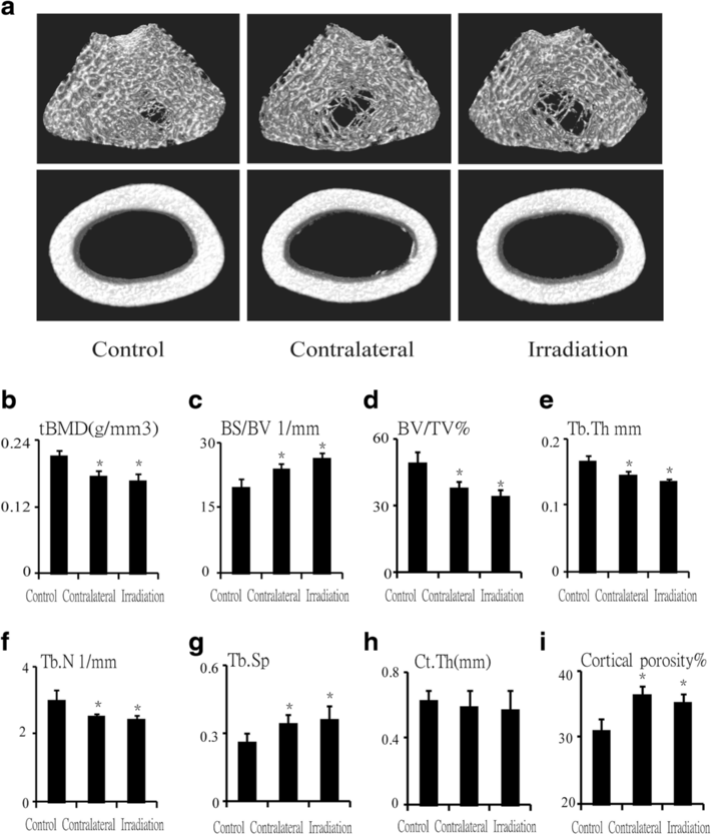
Effects of in vivo radiation exposure to single bone on bone microarchitecture in the femur 12 weeks post-irradiation. (**a**) Representative reconstructed images of μCT scans showing the trabecular and cortical bone at the femur and (**b**) tBMD. Differences in (**c**) BS/BV, (**d**) BV/TV, (**e**) Tb. Th, (**f**) Tb.N, and (**g**) Tb.Sp. The cortical bone in (**h**) Ct.Th. (**i**) Cortical porosity. Data were presented as means ± standard deviations, where *P < 0.05 (*n* = 16/group)

μCT was used to delineate a purely trabecular region of interest such as changes in bone volume and microarchitectural structure. Twelve weeks post-irradiation (20 Gy), trabecula BMD (tBMD) of the femur was reduced by 18.1 % (*P* < 0.05) at the contralateral femur and by 21.2 % (*P* < 0.05) at the irradiated femur relative to control (Fig. 1a, b); trabecular bone volume fraction (BV/TV) was reduced by 23.2 % (*P* < 0.05) at the contralateral femur and by 30.8 % (*P* < 0.05) at the irradiated femur relative to the control femur (Fig. 1d). The ratio of bone surface to the bone volume (BS/BV) was increased by 20.4 % (*P* < 0.05) at the contralateral femur and by 32.9 % (*P* < 0.05) at the irradiated femur compared to the control group (Fig. 1c). Trabecular thickness (Tb.Th) and trabecular number (Tb.N) were reduced significantly at 12 weeks after irradiation, Tb.Th was reduced by 12.1 % (*P* < 0.05) at the contralateral femur and by 17.5 % (*P* < 0.05) at the irradiated femur; meanwhile, Tb.N was reduced by 16.1 % (*P* < 0.05) at the contralateral femur and by 18.1 % (*P* < 0.05) at the irradiated femur relative to control (Fig. 1e, f). Trabecular separation (Tb.Sp) was increased sharply after irradiation by 31.9 % (*P* < 0.05) at the contralateral femur and by 39.0 % (*P* < 0.05) at the irradiated femur compared with control (Fig. 1g). Bone microarchitecture of the cortical femur changed slightly with no differences observed (Fig. 1a, h), but the cortical porosity increased by 17.9 % (*P* < 0.05) and 13.8 % (*P* < 0.05), respectively, at the contralateral and irradiated femur relative to control (Fig. 1i).

### The decreasing of bone mass and biomechanical quality after single radiation

**Fig. 2:**
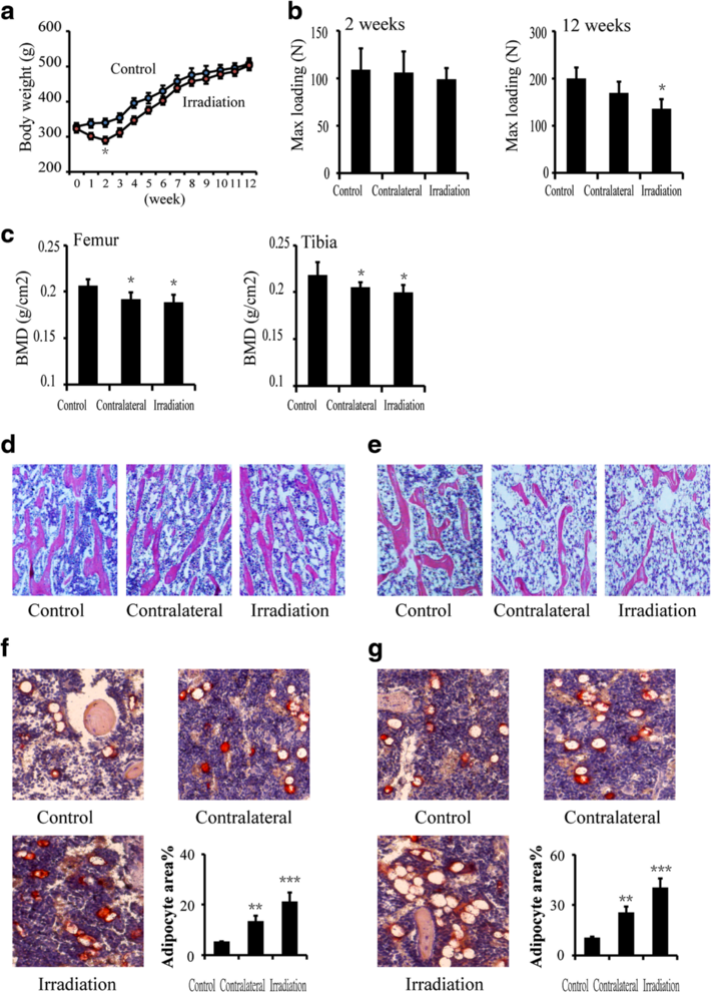
Effects of in vivo radiation exposure to single bone on (**a**) body weight, (**b**) the maximum loading of the femur at 2 weeks and at 12 weeks, (**c**) bone mineral density (BMD) of the femur and tibia, and (**d**, **e**) H&E sections of the tibia at 2 and 12 weeks are presented. (**f**, **g**) Oil red O sections of the tibia at 2 and 12 weeks are presented. Data were presented as means ± standard deviations, *P < 0.05, **P < 0.01 and ***P < 0.001 (*n* = 8/group)

One week after irradiation, body weight began to decline (−10.7 %, *P* > 0.05) and continued to decline rapidly (−20.7 %, *P* < 0.05) at 2 weeks after irradiation. However, at 3 weeks after irradiation, body weight increased, and no changes were observed in body weight between two groups since 5 weeks after irradiation (Fig. 2a). The max loading condition of the femur was slightly reduced at 2 weeks, but significantly reduced at 12 weeks after irradiation, and decreased by 15.6 % (*P* > 0.05) and 32.6 % (*P* < 0.05) at the contralateral and irradiated femur relative to the control femur (Fig. 2b). Bone mineral density (BMD) of the femur, determined by dual-energy X-ray absorptiometry (DXA), was reduced in the contralateral and irradiated femurs at 12 weeks post-irradiation and were significantly decreased by 7.1 and 8.8 % (*P* < 0.05), respectively, when compared to the control femur. BMD of the tibia was decreased by 6.0 and 8.5 % (*P* < 0.05), respectively, at the contralateral and irradiated tibia compared to the control tibia at 12 weeks after irradiation (Fig. 2c). The trabecular bone volume in the tibia, where no differences were observed at 2 weeks (Fig. 2d), decreased significantly in the contralateral and irradiated tibia at 12 weeks compared with control (Fig. 2e). The adipocyte area in the bone marrow of the contralateral and irradiated tibia increased significantly at 2 and 12 weeks after irradiation (Fig. 2f, g).

### The osteoblastogenesis decreased after single radiation

**Fig. 3:**
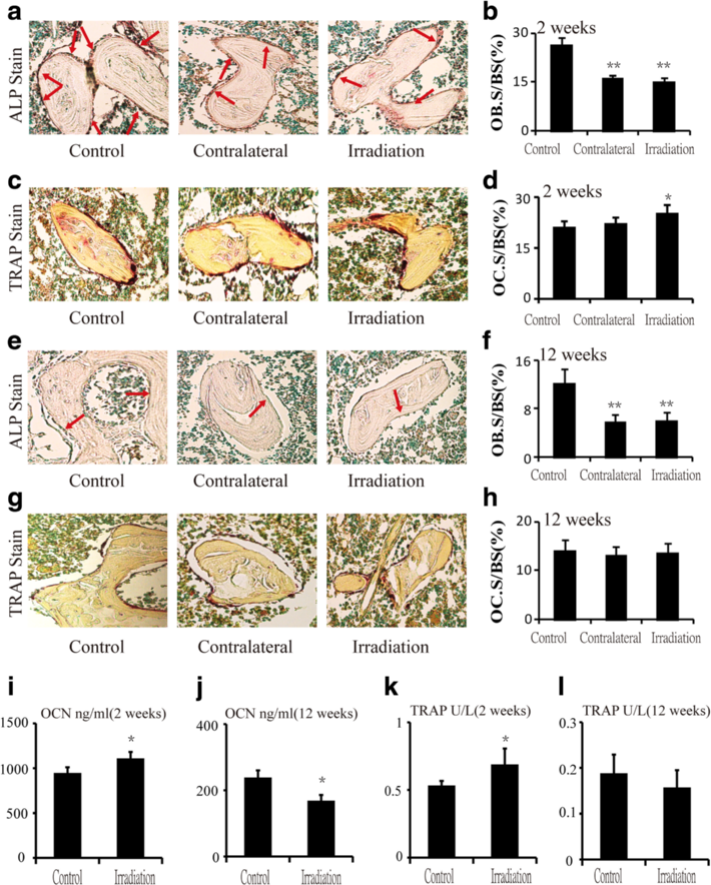
Effects of in vivo radiation exposure to single bone on (**a**) ALP-stained sections and (**b**) OB.S/BS at 2 weeks. (**c**) TRAP-stained sections and (**d**) OC.S/BS at 2 weeks. (**e**) ALP-stained sections and (**f**) OB.S/BS) at 12 weeks. (**g**) TRAP-stained sections and (**h**) OC.S/BS) at 12 weeks. Data were presented as means ± standard deviations, where *P < 0.05 and **P < 0.01 (*n* = 8/group). Serum bone marker changed at different time after irradiation. (**i**) Level of serum OCN at 2 weeks and (**j**) 12 weeks. (**k**) Level of serum TRAP at 2 weeks and (**l**) 12 weeks. Data were presented as means ± standard deviations, where *P < 0.05 (*n* = 16/group)

To examine the changes of osteoblast and osteoclast activity, histomorphometric analysis was performed on tartrate-resistant acid phosphatase (ALP) and tartrate-resistant acid phosphatase (TRAP)-stained sections. The results showed that the ALP-positive OB.S/BS was decreased by 38.5 % (*P* < 0.01) and 42.3 % (*P* < 0.01) in the contralateral and irradiated bone, respectively, relative to control after 2 weeks irradiation (Fig. 3a, b). After 12 weeks irradiation, the situation became worse, a 51.7 % (*P* < 0.01) reduction at contralateral and a 50.8 % (*P* < 0.01) reduction in irradiated rats (Fig. 3e, f). The TRAP-positive OC.S/BS was increased by 4.8 % (*P* > 0.05) at contralateral and by 19.0 % (*P* < 0.05) at irradiated group compared to control after 2 weeks (Fig. 3c, d). After 12 weeks post-irradiation, there were no differences between the two groups (Fig. 3g, h). ELISA analysis of bone turnover markers in serum revealed a time-dependent changes of the serum bone formation marker osteocalcin (OCN), a 17.3 % (*P* < 0.05) increase in rats at 2 weeks post-irradiation but a 29.9 % (*P* < 0.05) reduction in rats at 12 weeks post-irradiation (Fig. 3i, j). The serum bone resorption marker tartrate-resistant acid phosphatase 5b (TRAP5b) had the same trend with OCN—a 30.0 % (*P* < 0.05) increase in irradiated rats at 2 weeks post-irradiation and a 16.7 % (*P* > 0.05) reduction in rats at 12 weeks post-irradiation (Fig. 3k, l).

### The adipocyte differentiation of BMSCs increased after single radiation

**Fig. 4:**
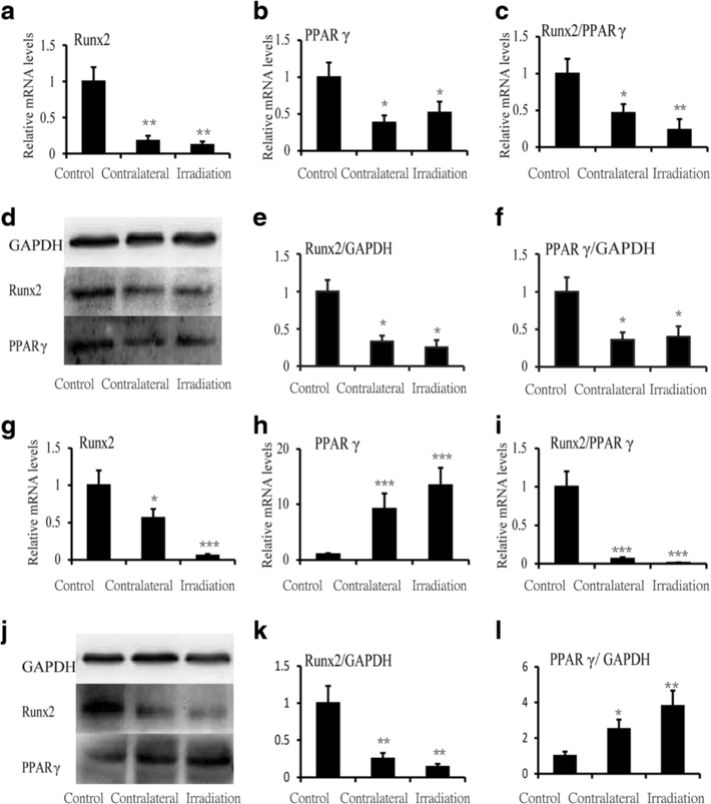
The relative mRNA and protein of BMSCs at 2 and 12 weeks after irradiation. The mRNA expression of (**a**) Runx2, (**b**) PPAR γ, and (**c**) the ratio of Runx2/PPAR γ at 2 weeks. The protein expression of (**e**) Runx2 and (**f**) PPAR γ at 2 weeks. The mRNA expression of (**g**) Runx2, (**h**) PPAR γ, and (**i**) the ratio of Runx2/ PPAR γ at 12 weeks. The protein expression of (**k**) Runx2 and (**l**) PPAR γ at 12 weeks. Data were presented as means ± standard deviations, where *P < 0.05, **P < 0.01, and ***P < 0.001 (*n* = 4/group)

To evaluate the influence of single irradiation on osteoblast/adipocyte differentiation of progenitors because of the sharply increase adipocytes in the bone marrow after irradiation, the expression of Runx2 and PPAR γ was determined in BMSCs at 2 and 12 weeks after irradiation by real-time PCR and Western blot. Results showed that the significant decrease of Runx2 (−82.3 % at contralateral, *P* < 0.01 and −88.0 % at irradiated, *P* < 0.01) and immediate decline of PPAR γ (−61.8 % at contralateral, *P* < 0.05 and −48.3 % at irradiated, *P* < 0.05) after 2 weeks post-irradiation (Fig. 4a, b), the ratio of Runx2/PPAR γ decreased by 53.6 % (*P* < 0.05) in contralateral rats and markedly decreased by 76.8 % (*P* < 0.01) in irradiated ones relative to control (Fig. 4c). The protein level of Runx2 and PPAR γ was similar to the changes of the messenger RNA (mRNA) expression in BMSCs (Fig. 4d–f). After 12 weeks irradiation, the expression of Runx2 continued to downregulate by 44.1 % (*P* < 0.05) and 94.5 % (*P* < 0.001) at contralateral and irradiated, respectively, but the expression of PPAR γ upregulated sharply about ninefold (*P* < 0.001) at contralateral and 13-fold (*P* < 0.001) at irradiated relative to control (Fig. 4g, h). Therefore, the ratio of Runx2/PPAR γ continued to sharply decrease with a 93.9 % (*P* < 0.001) reduction at contralateral and a 99.0 % (*P* < 0.001) reduction at irradiated (Fig. 4i). The protein level of Runx2 and PPAR γ was similar to the changes in the mRNA expression at 12 weeks after irradiation (Fig. 4j–l).

## Discussion

Retrospective study is a common method to examine the relationship between radiotherapy and the development of bone complications. Although the majority of these retrospective studies have identified bone complications following radiotherapy on the skeletal sites directly exposed to the radiation, bone complications are often found at sites without radiotherapy, which often occur later when the symptoms of acute irradiation have disappeared [18–20]. A randomized prospective study found that the high femoral fracture rate (6.7 % of a total 1716) in breast cancer patients receiving radiotherapy was not the consequence of the significant direct radiation exposure of the bone. However, the mechanism of local radiotherapy resulting in this abscopal effect on the non-irradiated skeleton remains unclear [21, 22].

Using a rat model, we have obtained evidence that local irradiation induces a time-dependent deterioration of both bone quantity (BMD) and quality (strength and microarchitecture) in the unexposed skeleton. This finding demonstrates that radiation damage to the bone does not require direct radiation exposure to the bone. Radiation-induced bone loss has historically been attributed to the long-term impairment of bone formation [23–28]. A rapid increase in osteoclastogenesis has been previously reported 1 week after body irradiation [29, 30]. Consistent with these reports, osteoclast number increased in the trabecular bone of our model at 2 weeks post-irradiation. Furthermore, the level of TRAP in the rat serum also increased after 2 weeks irradiation. Although osteoblast number in the trabecular bone decreased, the level of OCN in the serum was increased at 2 weeks post-irradiation, suggesting that radiation exposure did not compromise the coupling time of resorption and formation immediately after irradiation, so there were no obvious changes in the bone observed 2 weeks post-irradiation. This differed in comparison to the changes seen in some irradiation mouse models [29]. Interestingly, osteoclastogenesis in the irradiated rats was comparable to the control at 12 weeks post-irradiation. However, osteoblastogenesis in the trabecular bone was significantly suppressed with a reduced level of OCN in the serum 12 weeks post-irradiation. These results indicated that the balance between bone formation and bone resorption was altered, supporting the long-standing notion that ionizing radiation impairs bone formation in the late stage after irradiation [23–27, 30]. Additionally, a decrease in body weight was observed after local radiation, which was thought to be an acute irradiation effect of the stomach and intestine, leading to a reduction in body weight because of less feeding and loss of fat mass [31].

In addition to the downregulation of osteoblastic bone formation in vivo, dynamic bone slices revealed promotion of marrow adiposity, which was demonstrated by a continuous increase in the number of adipocytes after irradiation. A reciprocal relationship has been identified clinically between marrow adiposity and bone volume in cancer patients treated with radiotherapy [13, 32]. Consistent with these clinical findings, we reported a near 2.4-fold and 1.8-fold increase in marrow adiposity in the irradiated and contralateral tibia, respectively, 2 weeks after irradiation. Jia has previously reported rapid abscopal suppression of the bone marrow stromal cell population in mice after abdominal irradiation [33]. The bone marrow stroma hosts progenitors of bone-forming and bone-resorbing cells and regulates the survival and function of these progenitors and their progeny [34]. Despite no observed changes in osteoblast number after 2 weeks irradiation, the increase in adipocyte numbers might have exhausted the BMSC pool via an adipogenic lineage commitment switch, thus, leading to the decrease in osteoblast number observed in the current model at later time points. To further confirm this hypothesis, we detected the relative mRNA and protein expression levels of Runx2 and PPAR γ in BMSCs, which are important in determining the balance of BMSC differentiation into osteoblasts and adipocytes, respectively [35]. Runx2 is a crucial marker that determines the differentiation of BMSCs into osteoblasts and affects gene expression of downstream factors such as ALP, OCN, and osteopontin (OPN) [36]. PPAR γ regulates fatty acid storage, and the genes activated by PPAR γ stimulate lipid uptake and adipogenesis by fat cells. PPAR γ knockout mice fail to generate adipose tissue when fed a high-fat diet [37].

In the current model, the expression of Runx2 and PPAR γ was clearly downregulated 2 weeks post-irradiation and the ratio of Runx2/PPAR γ was also decreased. This was further changed at 12 weeks post-irradiation, whereby the expression of Runx2 was downregulated more significantly, the expression of PPAR γ was upregulated and the ratio of Runx2/PPAR γ decreased sharply. It is clear that this marrow defect is strongly associated with bone loss and might lead to increased fracture risk [18, 30, 38].

## Conclusions

To conclude, the etiology of radiation-induced skeletal complications appears multifactorial and time-dependent. In the current single femur and tibia irradiation model, osteoblast activation, decreased osteogenic potential, and increased marrow adiposity, might be critical for bone complications after irradiation. The reason why non-irradiated BMSCs demonstrate identical changes to those in the irradiated bone marrow is clearly of interest. Further studies are necessary to assess bone marrow changes at the cellular level and to examine the developmental influences of BMSCs after irradiation at both the irradiated and non-irradiated bone sites.

## Abbreviations

BS/BV: ratio of bone surface to the bone volume
BV/TV: bone volume fraction, ratio of the segmented bone volume to the total volume of the region of the region of interest
CtTh: average cortical thickness
PPAR γ: peroxisome proliferator-activated receptor γ
Runx2: runt-related transcription factor 2
Tb/Th: trabecular thickness, mean thickness of trabeculae
tBMD: trabecular bone mineral density
TbN: trabecular number, measure of the average number of trabeculae per unit length
TbSp: trabecular separation, mean distance between trabeculae
